# Hipertensos Tratados e Avaliados por Telemonitoramento Residencial da Pressão Arterial. Estudo TeleMRPA

**DOI:** 10.36660/abc.20200073

**Published:** 2021-05-21

**Authors:** Weimar Kunz Sebba Barroso, Audes Diógenes Magalhães Feitosa, Eduardo Costa Duarte Barbosa, Andréa Araujo Brandão, Roberto Dischinger Miranda, Priscila Valverde Oliveira Vitorino, Carlos Alberto Machado, Antônio Almeida Braga, Lúcio Paulo de Souza Ribeiro, Marco Antonio Mota-Gomes

**Affiliations:** 1 Universidade Federal de Goiás Goiânia GO Brasil Universidade Federal de Goiás - Liga de Hipertensão Arterial, Goiânia, GO - Brasil.; 2 Universidade Federal de Pernambuco Laboratório de Imunopatologia Keizo Asami Recife PE Brasil Laboratório de Imunopatologia Keizo Asami - Universidade Federal de Pernambuco - Clínica Medica, Recife, PE - Brasil.; 3 Instituto de Cardiologia Laboratório de Investigação Clínica Porto Alegre RS Brasil Instituto de Cardiologia - Laboratório de Investigação Clínica (LIC), Porto Alegre, RS - Brasil.; 4 Universidade do Estado do Rio de Janeiro Rio de Janeiro RJ Brasil Universidade do Estado do Rio de Janeiro, Rio de Janeiro, RJ - Brasil.; 5 Universidade Federal de São Paulo Escola Paulista de Medicina São Paulo SP Brasil Universidade Federal de São Paulo Escola Paulista de Medicina, São Paulo, SP - Brasil.; 6 Pontifícia Universidade Católica de Goiás Escola de Ciências Sociais e da Saúde Goiânia GO Brasil Escola de Ciências Sociais e da Saúde - Pontifícia Universidade Católica de Goiás, Goiânia, GO - Brasil.; 7 Estratégia de Saúde da Família Campos do Jordão SP Brasil Estratégia de Saúde da Família - Secretaria Municipal de Saúde Campos do Jordão, Campos do Jordão, SP - Brasil.; 8 Procape / MCor Recife PE Brasil Procape / MCor, Recife, PE - Brasil.; 9 Hospital do Coração Centro Universitário CESMAC Maceió AL Brasil Centro Universitário CESMAC - Hospital do Coração, Maceió, AL - Brasil.

**Keywords:** Hipertensão, Anti-Hipertensivos, Pressão Arterial, Monitoração Pressão Arterial Residência, TeleMRPA, Hipertensão do Jaleco Branco, Prevalência

## Abstract

**Fundamento:**

Hipertensos tratados avaliados apenas com a medida casual da pressão arterial (PA) podem estar sujeitos a decisões equivocadas.

**Objetivos:**

Avaliar o comportamento da PA pela medida casual e residencial (MRPA), o comportamento das classes de anti-hipertensivos e as prevalências de hipertensão do avental branco (HABNC) e mascarada não-controladas (HMNC).

**Métodos:**

Estudo transversal que avaliou pacientes pela plataforma TeleMRPA entre 2017 e 2019. Foram excluídos aqueles sem medicamentos, com 3 ou mais, em uso de espironolactona e alfa-2 agonistas. As variáveis analisadas foram: idade, sexo, índice de massa corporal (IMC), número de medidas válidas da PA, médias da PA sistólica (PAS) e diastólica (PAD) pela medida casual e MRPA, e as classes de anti-hipertensivos. Utilizados os testes *t* pareado e não pareado e qui-quadrado. Adotado nível de significância de 5%.

**Resultados:**

Selecionados 22.446 pacientes, dos quais 6.731 preencheram os critérios, sendo 61,3% do sexo feminino, com idade média de 57,8 (±12,6) anos e IMC médio de 29,0 (±5,1) kg/m^2^. Os valores médios de PAS e PAD foram 6,6 mmHg (p<0,001) e 4,4 mmHg (p<0,001) maiores na medida casual que na MRPA. As taxas de controle da PA foram de 57,0% pela medida casual e 61,3% pela MRPA (p<0,001), com prevalência de HABNC e HMNC de 15,4% e 11,1%, respectivamente. O bloqueio do sistema renina-angiotensina-aldosterona ocorreu em 74,6% das vezes e 54,8% estavam em monoterapia.

**Conclusões:**

O uso da MRPA deve ser considerado no acompanhamento de hipertensos tratados em virtude das elevadas prevalências de HABNC e HMNC. Os anti-hipertensivos tiveram comportamentos distintos nas medidas domiciliares. (Arq Bras Cardiol. 2021; [online].ahead print, PP.0-0)

## Introdução

A medida casual da pressão arterial (PA) em pacientes hipertensos, seja para diagnóstico ou avaliação do controle, apresenta fragilidades importantes e pode levar a equívocos de interpretação.^1^^,^^2^

Por outro lado, o tratamento farmacológico da hipertensão arterial (HAS) e a consequente redução das cifras pressóricas são capazes de diminuir de forma significativa a incidência dos principais desfechos cardiovasculares.^3^^,^^4^ Apesar desse conhecimento, a grande maioria dos dados epidemiológicos relativos à prevalência de HAS em nosso país deriva de questionários epidemiológicos, como a Pesquisa Nacional de Saúde (21,4%)^5^ e o VIGITEL (24,7%),^6^ sendo os dados relativos ao controle, em quase sua totalidade, obtidos a partir da medida casual.^7^

Portanto, é importante que a avaliação da eficácia dos anti-hipertensivos e do controle adequado da PA em pacientes tratados seja feita por métodos com maior acurácia que a medida casual, podendo-se utilizar para essa finalidade a monitorização ambulatorial (MAPA) ou residencial da pressão arterial (MRPA).^8^^–^^10^

Este é o primeiro estudo em um grande número de hipertensos tratados com diferentes classes de anti-hipertensivos e avaliados por método de MAPA e telemedicina com o objetivo de investigar se os valores no consultório e no domicílio apresentam diferenças, se as diferentes classes de anti-hipertensivos têm comportamentos distintos quando avaliadas no domicílio do paciente e quais as prevalências de hipertensão do avental branco não controlada (HABNC) e de hipertensão mascarada não controlada (HMNC).

## Métodos

Este estudo foi submetido ao Comitê de Ética em Pesquisa Humana do Hospital das Clínicas da Universidade Federal de Goiás sob o número CAEE 99691018.7.0000.5078, tendo sido aprovado.

Estudo transversal, que avaliou pacientes que realizaram exames na plataforma TeleMRPA (www.telemrpa.com) de maio de 2017 a setembro de 2019. A plataforma foi desenvolvida como ferramenta de laudo a distância por telemonitoramento com características que permitem a análise e o filtro do banco de dados de acordo com as perguntas científicas que se pretendem investigar. O algoritmo matemático utilizado possibilita a análise com proteção dos dados pessoais do paciente, assim como das clínicas ou unidades de saúde, seja para a interpretação do exame, seja para a construção de projetos de pesquisa. Por não se tratar de um software e sim de uma plataforma acessível em qualquer terminal de computador, tablet ou smartphone, a inserção dos dados relativos à medida da PA pode ser feita de forma remota e simplificada.

**Figura 1:**
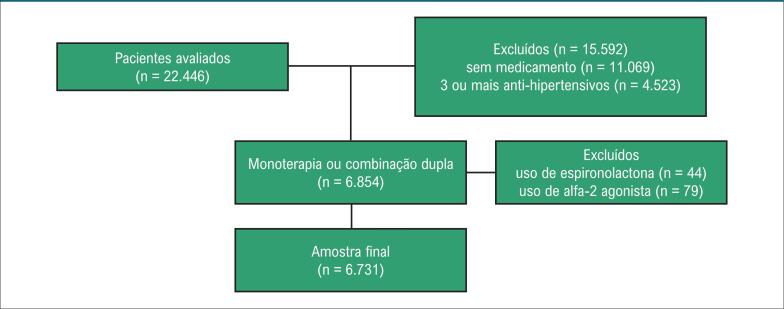
*Fluxograma de seleção da amostra do estudo*.

Foram incluídos neste estudo pacientes com idade mínima de 18 anos, em uso de fármacos anti-hipertensivos e que apresentavam como estratégia de tratamento monoterapia ou combinação com duas classes distintas. Foram excluídos pacientes que não utilizavam anti-hipertensivos, em uso de combinação de três ou mais anti-hipertensivos e ainda aqueles que utilizavam espironolactona e alfa-2 agonista como monoterapia (Figura 1).

Fonte: os autores.

Foram utilizados os seguintes dados da plataforma TeleMRPA: sexo (masculino/feminino); idade (em anos calculada a partir da data de nascimento); índice de massa corporal (IMC); número de medidas válidas da MRPA; PA sistólica (PAS) e diastólica (PAD) obtidas pela MRPA e de forma casual; e classe dos medicamentos utilizados.

**Figura 2:**
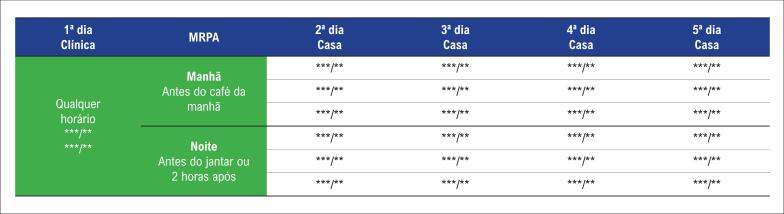
Protocolo de monitorização residencial de pressão arterial (MRPA) de acordo com a diretriz brasileira (***/**: medição da pressão arterial). ^8^ ^,^ ^9^

Para o cálculo do IMC, foram utilizados o peso e a altura aferidos e a fórmula de Quetelet.^11^ Para a medida da MRPA, o aparelho foi disponibilizado para o paciente, que foi orientado sobre manuseio e técnica adequados para a medida da PA no dia da entrega do mesmo.^3^ Ainda nesse primeiro dia, foram realizadas duas medidas no ambiente da clínica/consultório e, nos quatro dias subsequentes, o paciente (e/ou cuidador/acompanhante) realizou as medidas em seu domicílio conforme o protocolo. Considerou-se como medida casual a média das duas medidas do primeiro dia e como medida domiciliar a média das 24 medidas do segundo ao quinto dia (Figura 2).^12^^,^^13^

Foram utilizados aparelhos automáticos validados das marcas Omron, Geratherm e Microlife.

Os dados foram exportados da plataforma TeleMRPA para o excel. Todas as classes de medicamentos descritas na plataforma foram revisadas e codificadas por duas equipes de trabalho. Em seguida, os bancos de dados foram cruzados para identificação de discrepâncias que, quando identificadas, foram revisadas com toda a equipe e a coordenação.

### Análise estatística

Para a análise dos dados foi utilizado o *software Stata,* versão 14.0. Foi empregada estatística descritiva com utilização de médias e desvio-padrão para as variáveis contínuas com distribuição normal e de frequências absoluta e relativa para as variáveis categóricas. Foi aplicado o teste de Kolmogorov-Smirnov para verificação da distribuição de dados das variáveis. A comparação entre os valores de PA obtidos por meio da MRPA e da medida casual foi realizada com o teste *t* pareado. Para a comparação dos valores de PA de acordo com o medicamento, foi utilizado o teste *t* não pareado. Para comparações entre os pacientes que atingiram e não atingiram as metas segundo a medida casual e a MRPA, foi utilizado o teste qui-quadrado. Para todas as análises, foi adotado um nível de significância de 5%.

## Resultados

Foram avaliados 6.731 pacientes, 61,3% do sexo feminino, com idade média de 57,8 (±12,6) anos e IMC médio de 29,0 (±5,1) kg/m^2^. Os valores da PA casual foram maiores que os do domicílio e esse comportamento se repetiu com o uso de monoterapia ou combinação de fármacos, bem como com todas as classes de medicamentos (Tabela 1). O número médio de medidas válidas/exame foi de 23,5 (±1,6). As diferenças nos valores médios da PAS e PAD foram de 6,6 mmHg (p<0,001) e 4,4 mmHg (p<0,001), respectivamente. Essas diferenças caracterizam o efeito do avental branco e se mantiveram com significância estatística em todas as estratégias de tratamento.

**Tabela 1:** Descrição da pressão arterial da amostra segundo a utilização de medicamento e comparação entre a pressão arterial pela medida casual e pela monitorização residencial da pressão arterial (MRPA) total e segundo a estratégia medicamentosa, n= 6.731

Variável	n	%	MRPA	Casual	p
**Total**					
PAS	6.731	100	126,2 ± 15,3	132,8 ± 19,6	<0,001
PAD			79,6 ± 9,7	84,0 ± 11,6	<0,001
**BRA**					
PAS	2.254	33,5	127,1 ± 15,2	133,5 ± 19,2	<0,001
PAD			80,4 ± 9,6	84,9 ± 11,6	<0,001
**IECA**					
PAS	595	8,8	124,7 ± 14,2	130,2 ± 18,1	<0,001
PAD			79,3 ± 9,0	83,0 ± 10,5	<0,001
**ACC**					
PAS	196	2,9	127,2 ± 13,1	134,0 ± 17,4	<0,001
PAD			80,2 ± 9,5	84,4 ± 11,2	<0,001
**Diurético**					
PAS	173	2,6	123,1 ± 13,8	132,2 ± 19,1	<0,001
PAD			79,1 ± 9,3	85,5 ± 10,6	<0,001
**Betabloqueador**					
PAS	474	7,0	123,2 ± 15,2	130,6 ± 19,4	<0,001
PAD			77,7 ± 10,0	82,4 ± 11,2	<0,001
**BRA + ACC**					
PAS	683	10,1	127,1 ± 14,6	133,6 ± 19,1	<0,001
PAD			79,0 ± 9,7	83,2 ± 11,8	<0,001
**IECA + ACC**					
PAS	332	4,9	126,2 ± 12,6	132,4 ± 16,3	<0,001
PAD			79,9 ± 8,9	83,8 ± 10,4	<0,001
**BRA + diurético**					
PAS	1.015	15,1	125,2 ± 16,2	132,8 ± 21,1	<0,001
PAD			79,6 ± 9,6	84,5 ± 12,1	<0,001
**IECA + diurético**					
PAS	151	2,2	124,6 ± 15,8	132,7 ± 20,0	<0,001
PAD			78,8 ± 9,5	84,0 ± 11,1	<0,001
**Betabloqueador + IECA**					
PAS	134	2,1	127,6 ± 17,1	133,9 ± 21,9	<0,001
PAD			79,0 ± 10,4	82,4 ± 13,2	<0,001
**Betabloqueador + BRA**					
PAS	475	7,1	129,5 ± 18,0	135,5 ± 22,3	<0,001
PAD			79,1 ± 10,6	83,0 ± 12,7	<0,001
**Betabloqueador + diurético**					
PAS	137	2,0	122,0 ± 14,9	130,9 ± 21,0	<0,001
PAD			78,1 ± 8,3	84,0 ± 11,3	<0,001
**Betabloqueador + ACC**					
PAS	65	1,0	125,5 ± 16,4	131,9 ± 21,6	<0,001
PAD			77,4 ± 10,7	82,1 ± 12,1	<0,001
**ACC + diurético**					
PAS	45	0,7	130,8 ± 14,6	137,1 ± 18,6	<0,001
PAD			81,7 ± 11,0	86,3 ± 13,1	<0,001

*Teste t pareado. BRA: bloqueador do receptor de angiotensina; IECA: inibidor da enzima conversora de angiotensina; ACC: antagonista de canal de cálcio; PAS: pressão arterial sistólica; PAD: pressão arterial diastólica. Fonte: os autores*.

Em relação às preferências de estratégia medicamentosa, 54,8% dos pacientes estavam em uso de monoterapia e 45,2%, de combinações duplas. O bloqueio do sistema renina-angiotensina-aldosterona (SRAA) foi a opção mais frequente, sendo que 58,7% dos pacientes estavam em uso de bloqueadores dos receptores de angiotensina (BRA) e 15,9%, de inibidores da enzima conversora de angiotensina (IECA) (Tabela 1).

Quando avaliamos o controle da PA de acordo com as metas ‘menor que 140 mmHg e 90 mmHg’ para a medida casual e ‘menor que 135 mmHg e 85 mmHg’ para a MRPA, conforme diretrizes vigentes,^3^^,^^4^ encontramos taxas de 57,0% e 61,3%, respectivamente (p<0,001). A prevalência de HABNC foi 15,4% e de HMNC, 11,1% (Tabela 2).

**Tabela 2:** Controle da pressão arterial avaliada pela medida casual (<140 e <90 mmHg) e pela monitorização residencial da pressão arterial (MRPA: <135 e <85 mmHg), n= 6.731

	Meta pela medida casual		Total	
	**<140 e <90mmHg**	**≥ 140 e/ou ≥ 90 mmHg**		
Meta pela MRPA				
<135 e <85 mmHg	3.093 (45,9%)	1.034 (15,4%)*	4.127 (61,3%)	p< 0,001
≥135 e/ou ≥85 mmHg	744,9 (11,1%)†	1.860 (27,6%)	2.604 (38,7%)	
Total	3.837 (57,0%)	2.894 (43,0%)	6.731 (100,0%)	

*Qui quadrado.*

* *hipertensão do avental branco não controlada,*

† *hipertensão mascarada não controlada. Fonte: os autores*

As comparações entre as diferentes classes de anti-hipertensivos são mostradas na Tabela 3 (somente monoterapia) e na Tabela 4 (somente combinações duplas) para a medida da PA pela MRPA.

**Tabela 3:** Valores de significância (p) referentes às comparações das pressões arteriais sistólica e diastólica obtidas pela MRPA, segundo diferentes classes de anti-hipertensivos em monoterapia, n= 6.731

Medicamentos					
	**Medicamento (comparações PAS)**	IECA	ACC	DIUR	BB
BRA		**<0,01**	0,987	**<0,001**	**<0,001**
IECA		-	**0,035**	0,060	0,095
ACC		-	-	**<0,001**	**0,002**
DIUR		-	-	-	0,630
BB		-	-	-	-
	**Medicamento (comparações PAD)**	IECA	ACC	DIUR	BB
BRA		**0,009**	0,737	**<0,001**	**<0,001**
IECA		-	0,231	0,115	0,005
ACC		-	-	**0,028**	**0,002**
DIUR		-	-	-	0,557

*Teste t não pareado. BRA: bloqueador do receptor de angiotensina; IECA: inibidor da enzima conversora de angiotensina; ACC: antagonista de canal de cálcio; BB: betabloqueador; PAS: pressão arterial sistólica; PAD: pressão arterial diastólica. Fonte: os autores.*

**Tabela 4:** Valores de significância (p) referentes à comparação das pressões arteriais sistólica e diastólica obtidas pela MRPA segundo diferentes combinações de dois fármacos anti-hipertensivos, n= 6.731

Combinação dupla de medicamentos								
Comparação PAS	IECA com ACC	BRA com DIUR	IECA com DIUR	BB com IECA	BB com BRA	BB com DIUR	BB com ACC	ACC com DIUR
BRA com ACC	0,358	**0,018**	0,070	0,713	0,012	<0,001	0,419	0,099
IECA com ACC	-	0,317	0,239	0,332	**0,004**	**0,001**	0,696	**0,023**
BRA com DIUR	-	-	0,671	0,117	**<0,001**	**0,026**	0,894	**0,024**
IECA com DIUR	-	-	-	0,132	**0,003**	0,144	0,714	0,021
BB com IECA	-	-	-	-	0,276	0,004	0,417	0,263
BB com BRA	-	-	-	-	-	**<0,001**	0,093	0,641
BB com DIUR	-	-	-	-	-	-	0,129	<0,001
BB com ACC	-	-	-	-	-	-	-	0,087

Comparação PAD	IECA com ACC	BRA com DIUR	IECA com DIUR	BB com IECA	BB com BRA	BB com DIUR	BB com ACC	ACC com DIUR
BRA com ACC	0,133	0,195	0,800	0,995	0,842	0,270	0,204	0,077
IECA com ACC	-	0,581	0,190	0,319	0,243	**0,030**	**0,040**	0,239
BRA com DIUR	-	-	0,317	0,485	0,366	0,065	0,072	0,166
IECA com DIUR	-	-	-	0,856	0,725	0,475	0,343	0,087
BB com IECA	-	-	-	-	0,903	0,397	0,313	0,145
BB com BRA	-	-	-	-	-	0,264	0,216	0,125
BB com DIUR	-	-	-	-	-	-	0,644	**0,020**
BB com ACC	-	-	-	-	-	-	-	**0,043**

*Teste t não pareado. BRA: bloqueador do receptor de angiotensina; IECA: inibidor da enzima conversora de angiotensina; ACC: antagonista de canal de cálcio; BB: betabloqueador; PAS: pressão arterial sistólica; PAD: pressão arterial diastólica. Fonte: os autores.*

## Discussão

O presente estudo contribui com a análise na prática clínica de número expressivo de pacientes hipertensos tratados com medicamentos, avaliados pela medida casual da PA e pela MRPA. Os achados confirmam que menores médias de PA são observadas na MRPA, independentemente do uso de monoterapia ou combinação de fármacos, bem como das classes de anti-hipertensivos prescritas. Observaram-se alta taxa de controle da PA, maior com a MRPA, e prevalências relevantes de HABNC e HMNC, o que tem implicações prognósticas potenciais e reforça a necessidade do uso da medida da PA fora do consultório como parâmetro para adequada abordagem e seguimento do paciente hipertenso.

Em relação às características da amostra estudada, é importante ressaltar que se trata de uma população com idade média próxima a 60 anos e IMC aumentado, aspectos relevantes em virtude de serem fatores que tornam o tratamento da HAS e o seu controle mais difíceis.^14^^–^^16^

Outro ponto importante é que, apesar de as últimas diretrizes nacionais e internacionais recomendarem a combinação de fármacos como uma estratégia preferencial para a maioria dos pacientes hipertensos, ainda observamos que 54,8% de nossa amostra estava em uso de monoterapia.^3^^,^^4^^,^^17^^,^^18^ Na análise das classes de medicamentos utilizadas fica nítida a preferência pela estratégia de bloquear o SRAA, que aconteceu em 74,6% dos pacientes, sendo que a frequência de uso dos BRA foi 3,7 vezes maior que a dos IECA. Outras classes de anti-hipertensivos tiveram uma frequência baixa de uso em monoterapia. As combinações de BRA ou IECA com diuréticos (DIUR) ou antagonistas de canal de cálcio (ACC) foram preferidas, o que está bem alinhado com as recomendações atuais.^19^^,^^20^

Os valores de PAS e PAD apresentaram-se sempre mais altos e com significância estatística nas medidas no consultório comparadas às domiciliares, independentemente da classe de anti-hipertensivos e da estratégia de monoterapia ou combinação. Em média, as diferenças foram de 6,6 mmHg para PAS (p<0,001) e de 4,4 mmHg para a PAD (p<0,001) e se repetiram com maior ou menor intensidade em todas as medicações utilizadas. Esse achado remete à necessidade de considerarmos o cenário de HABNC como um fenótipo frequente em pacientes tratados e que, quando presente, pode induzir à utilização de medicamentos em quantidade ou doses maiores que o necessário quando avaliamos os hipertensos tratados apenas com base nas medidas de consultório.^2^^,^^8^^,^^9^^,^^21^ Em nossa amostra, a prevalência de HABNC foi 15,4% e a de HMNC, 11,1%, significando erro da interpretação de controle adequado com a estratégia terapêutica de 26,5%.

A HABNC torna-se ainda mais relevante se considerarmos que, sob o olhar da MRPA, temos mais pacientes na meta do que quando avaliados pela medida casual (61,3% vs 57%, p<0,001), novamente reforçando a tese de que, com as medidas domiciliares, poderemos fazer ajustes mais adequados ao comportamento da PA no dia a dia do paciente. Vale considerar ainda que, diferentemente do que se imaginava, a HABNC pode ser encontrada tanto quando temos elevações do componente sistólico quanto diastólico pela medida casual, e em todas as faixas etárias.^21^ Outro fenótipo a ser considerado é o da HMNC, que ainda é uma das grandes dúvidas no cenário do tratamento da HAS: como lidar com pacientes com a PA controlada no consultório e elevada nas medidas domiciliares e quais seriam as metas pela MRPA?^22^

Nas comparações das classes de anti-hipertensivos, encontramos diferenças significativas no que tange à redução dos valores pressóricos. Entretanto, devemos considerar como fator limitante o fato de que, como este estudo não foi randomizado para esse tipo de comparação em relação às classes, às doses utilizadas e às características dos pacientes, essas diferenças devem ser analisadas com cautela. Esses achados são coincidentes com os de outras publicações, principalmente metanálises de estudos randomizados, que já haviam descrito diferenças em termos de potência dependentes das doses utilizadas e indicações, porém através da análise de medida casual da PA.^23^^–^^25^ No presente estudo, as diferentes classes de anti-hipertensivos parecem apresentar potências distintas, quando avaliadas pela MRPA, o que reforça a necessidade de individualização do paciente na escolha da melhor estratégia para o tratamento.

A nosso ver, o aspecto relevante é a maior prevalência do uso dos BRA como estratégia de bloqueio do SRAA e sua combinação com ACC e DIUR. É possível que a preferência pelo bloqueio do SRAA como estratégia de tratamento da HAS tenha respaldo na literatura que demonstra evidências de redução expressiva da PA, proteção cardiovascular e baixa incidência de efeitos colaterais com essa classe de fármacos, seja em monoterapia seja em combinação.^26^^,^^27^

## Conclusões

Em hipertensos tratados, a análise do controle adequado da PA deveria ser baseada não somente nos valores do consultório como também nas medidas domiciliares.

As diferentes classes de anti-hipertensivos apresentaram comportamentos distintos em relação à redução da PA mesmo quando avaliadas com a MRPA e esse achado deve ser mais bem investigado com estudos randomizados prospectivos.

A elevada prevalência de HABNC e HMNC sugere que, quando a decisão terapêutica é baseada apenas nas medidas obtidas em consultório, podemos adotar condutas inadequadas, o que tem potencial impacto na abordagem e no seguimento dos pacientes hipertensos.

### Coinvestigadores nacionais

Adriana Camargo Oliveira (Goiânia, GO), Adriana Siqueira S. Menezes (Recife, PE), Ana Paula Nana P Martingo (Sorocaba, SP), Anderson da Costa Armstrong (Petrolina, PE), Anderson Daniel C. Rodrigues (São José dos Campos, SP), Andrea Garcez R. Dias (Rio de Janeiro, RJ), André K. Vidigal de Vasconcellos (Caruaru, PE), Ângela Maria F. Lima (Gravataí, RS), Annelise Machado Gomes de Paiva (Maceió, AL), Anselmo Honorato S. Souza (Goiânia, GO), Antônio Almeida Braga (Recife, PE), Anyotan Cruz Nascimento (Americana, SP), Aristeu Haroldo K. Mizuta (São José dos Campos, SP), Breno Gontijo de Camargos (Taguatinga, DF), Bruno Alencar Fonseca (Belo Horizonte, MG), Bruno Augusto A. Nogueira (São José dos Campos, SP), Bruno Daniel Ferrari (Assis, SP), Bruno José P. Coutinho (Carpina, PE), Carlos Alberto Machado (Campos do Jordão, SP), Carlo Bonasso (São Paulo, SP), Carlo Bonasso Filho (São Paulo, SP), Carlos Filinto de Almeida (Campo Grande, MS), Carolina Paes B. Saturnino Braga (Rio de Janeiro, RJ), César Lourenço Nezello (Lagoa Vermelha, RS), Claudinelli Alvarenga Aguilar (Goiânia, GO), Cristiane Bueno de Souza (Campos do Jordão, SP), Daniella Rosano Gregorini (Jundiaí, SP), Danielle Batista Leite (Fernando Figueira, PE), Eduardo Azevedo Junior (Cabo Frio, RJ), Eduardo Siqueira (Mogi das Cruzes, SP), Eduardo Zen (Curitiba, PR), Elder Gil Alves da Cruz (Salgueiro, PE), Eliel Barreto César (Americana, SP), Erika Maria Gonçalves Campana (São Gonçalo, RJ), Esther G.D. Lima de Barros Carvalho (Guarabira, PB), Euler Manenti (Porto Alegre, RS), Fabiano de Souza Ramos (Nova Iguaçu, RJ), Fábio Argenta (Cuiabá, MT), Fábio Serra Silveira (Aracaju, SE), Fernando Alfredo Fonseca (Petrópolis, RJ), Fernando Pivatto (Porto Alegre, RS), Flávia Karina S. Oliveira (São José dos Campos, SP), Flávio Henrique A. Pires Véras (Mossoró, RN), Francisco Deoclécio Pinheiro (Itapipoca, CE), Giovanni Alves Saraiva (Olinda, PE), Guilhermo Hinestrosa (São Paulo, SP), Gustavo A.C.S. Barros (Recife, PE), Gustavo Costa Motta (Santa Maria, RS), Hiran de Paula (Itabaiana, SE), Humberto Graner Moreira (Goiânia, GO), Idália de Sousa Andrade (São Paulo, SP), Ivaldo Calado (Recife, PE), Jadil Francisco Fusturath Jr (Porto Velho, RO), Joaquim Carlos P. Sales (Ribeirão Preto, SP), João Felix Morais Filho (Natal, RN), João Francisco M. Pacheco (Belém, PA), João Henrique R. Ferreira (Gama, DF), Jonathan Scapin Zagatti (Jales, SP), José Roberto B. Leal Filho (Caruaru, PE), Josafá de Oliveira Costa (Igarassu, PE), José Roberto Moya (Cuiabá, MT), José Wladimir Tambelli Pires (Itapetinga, SP), Josiedson Pontes de Farias (Caruaru, PE), Leonardo da Costa Velasco (Campos dos Goytacazes, RJ), Lilian Mesquita (Rio de Janeiro, RJ), Lola Santos F. Quinta (Goiânia, GO), Lorena Maia (Goiânia, GO), Luam Vieira de Almeida Diógenes (Teresina, PI), Lúcia Cristina F. Lenzi (Rio de Janeiro, RJ), Luís Fernando M. Mandrá (Americana, SP), Luiz Kencis Junior (São Paulo, SP), Luiz Fernando de Oliveira Urzeda (Goiânia, GO), Marcelo De Carli Cavalcanti (Petrolina, PE), Marcelo Júlio de Oliveira (Ribeirão Preto, SP), Marco Antônio Alves (Escada, PE), Marco Antônio C. Peralva (Juiz de Fora, MG), Maria Beatriz M.B. Rodrigues (Porto Velho, RO), Maria Christina C. Ballut (Manaus, AM), Murillo Antunes (Bragança Paulista, SP), Nelson Dinamarco (Ilhéus, BA), Nildo Magalhães (Jundiaí, SP), Otacílio Gomes de Oliveira Filho (Sete Lagoas, MG), Paulo E.F. Ortiz Júnior (Porto Alegre, RS), Paulo Meirelles (Salvador, BA), Paulo Roberto P. Sant'Ana (Nova Iguaçu, RJ), Paulo Sergio L. Soares (Vassouras, RJ), Pedro Guimarães M. Silva (Goiânia, GO), Rafael Nogueira de Macedo (Fortaleza, CE), Rafael Santos Costa (Nova Iguaçu, RJ), Reginaldo Peixoto de Melo Neto (Recife, PE), Roberto Dultra (Itabuna, BA), Robson Pierre Pacífico A. Filho (Goiânia, GO), Rodrigo Cunha (Uberaba, MG), Rogério Krakauer (Santo André, SP), Rogério M. Ruiz (São Paulo, SP), Rosa Maria Rondon (Campo Grande, MS), Tássia Tâmara Silva Feitosa (Recife, PE), Rui Morando (Americana, SP), Tsuneo Antônio Alberto Goto (Guarulhos, SP), Valéria Tatyane de Rezende (Goiânia, GO), Vanderlei Magalhães da Silveira (Passo Fundo, RS), Vanildo S. Guimarães Neto (Recife, PE), Victoria Alves Melo (Goiânia, GO), Vilma Helena Burlamaqui (Niterói, RJ), Wenderson Tavares dos Santos (Belo Horizonte, MG).
