# Supplementary material for: Hipertensos Tratados e Avaliados por Telemonitoramento Residencial da Pressão Arterial. Estudo TeleMRPA
Source: Arq Bras Cardiol. 2021 May 21;117(3):520–7. [Article in Portuguese] doi: 10.36660/abc.20200073 (PMC8462946; doi:10.36660/abc.20200073)
Supplement: Translation

# Translation: Treated Hypertensive Patients Assessed by Home Blood Pressure Telemonitoring. TeleMRPA Study

**Authors:** Weimar Kunz Sebba Barroso, Audes Diógenes Magalhães Feitosa, Eduardo Costa Duarte Barbosa, Andréa Araujo Brandão, Roberto Dischinger Miranda, Priscila Valverde Oliveira Vitorino, Carlos Alberto Machado, Antônio Almeida Braga, Lúcio Paulo de Souza Ribeiro, Marco Antonio Mota-Gomes

**Affiliations:** 1 Goiânia GO Brazil Universidade Federal de Goiás - Liga de Hipertensão Arterial, Goiânia, GO - Brazil.; 2 Recife PE Brazil Laboratório de Imunopatologia Keizo Asami - Universidade Federal de Pernambuco - Clínica Medica, Recife, PE - Brazil.; 3 Porto Alegre RS Brazil Instituto de Cardiologia - Laboratório de Investigação Clínica (LIC), Porto Alegre, RS - Brazil.; 4 Rio de Janeiro RJ Brazil Universidade do Estado do Rio de Janeiro, Rio de Janeiro, RJ - Brazil.; 5 São Paulo SP Brazil Universidade Federal de São Paulo Escola Paulista de Medicina, São Paulo, SP - Brazil.; 6 Goiânia GO Brazil Escola de Ciências Sociais e da Saúde - Pontifícia Universidade Católica de Goiás, Goiânia, GO - Brazil.; 7 Campos do Jordão SP Brazil Estratégia de Saúde da Família - Secretaria Municipal de Saúde Campos do Jordão, Campos do Jordão, SP - Brazil.; 8 Recife PE Brazil Procape / MCor, Recife, PE - Brazil.; 9 Maceió AL Brazil Centro Universitário CESMAC - Hospital do Coração, Maceió, AL - Brazil.

**Keywords:** Hypertension, Antihypertensive Agents, Blood Pressure, Study Blood Pressure Monitoring Home, TeleMRPA, Prevalence

## Abstract

**Background:**

Hypertensive patients undergoing treatment and assessed only by casual blood pressure (BP) measurement may be subject to mistaken decisions.

**Objective:**

To assess BP behavior by measuring its levels at the office (casual) and at home (HBPM), the behavior of different classes of antihypertensive drugs, and the prevalence of uncontrolled white-coat hypertension (UCWCH) and uncontrolled masked hypertension (UCMH).

**Methods:**

Cross-sectional study assessing patients who underwent BP monitoring in the TeleMRPA platform between 2017 and 2019. The exclusion criteria were: use of no antihypertensive drug; combined use of 3 or more antihypertensive drugs; and use of spironolactone and alpha-2 agonist. The variables analyzed were: age, sex, body mass index (BMI), number of valid BP measurements, means of systolic and diastolic blood pressure (SBP and DBP, respectively) obtained from HBPM and casual measurement, and the classes of antihypertensive drugs. Paired and unpaired t tests, as well as chi-square test, were used. The 5% significance level was adopted.

**Results:**

This study selected 22 446 patients, 6731 of whom met the inclusion criteria [61.3%, female sex; mean age, 57.8 (±12.6) years; mean BMI, 29.0 (±5.1) kg/m^2^]. Mean SBP and DBP were 6.6 mm Hg (p<0.001) and 4.4 mm Hg (p<0.001) higher in casual measurement than in HBPM. The rates of BP control were 57.0% in casual measurement and 61.3% in HBPM (p<0.001), and the prevalence of UCWCH and UCMH was 15.4% and 11.1%, respectively. Renin-angiotensin-aldosterone system blockade was observed in 74.6% of the patients, and 54.8% were on single-drug therapy.

**Conclusions:**

HBPM should be considered for the follow-up of treated hypertensive patients because of the high prevalence of UCWCH and UCMH. Antihypertensive drugs behaved differently in HBPM. (Arq Bras Cardiol. 2021; [online].ahead print, PP.0-0)

## Introduction

Casual blood pressure (BP) measurement of hypertensive patients for both diagnosis and control assessment has important flaws and can lead to misinterpretation.^1^^,^^2^

The pharmacological treatment of systemic arterial hypertension (SAH) and the consequent reduction in BP levels are known to significantly decrease the incidence of major cardiovascular outcomes.^3^^,^^4^ Nevertheless, most epidemiological data regarding the prevalence of SAH in Brazil derive from epidemiological questionnaires, such as the Brazilian Health Survey (*Pesquisa Nacional de Saúde* – 21.4%)^5^ and VIGITEL (24.7%),^6^ and most data concerning BP control have been obtained via casual measurement.^7^

Therefore, the efficacy of antihypertensive drugs and proper BP control of patients undergoing treatment should be assessed by using more accurate methods than casual measurement, such as ambulatory BP monitoring (ABPM) or home BP monitoring (HBPM).^8^^–^^10^

This is the first study with many hypertensive patients treated with different classes of antihypertensive drugs and assessed by use of ABPM and telemedicine, aiming at investigating: whether the BP levels measured at the doctor's office and at home differ; whether the different classes of antihypertensive drugs behave differently when BP is measured at home; and the prevalence of uncontrolled white-coat hypertension (UCWCH) and of uncontrolled masked hypertension (UCMH).

## Methods

This study was submitted to the Committee on Ethics and Research of the Hospital das Clínicas da Universidade Federal de Goiás (# CAEE 99691018.7.0000.5078) and was approved.

This is a cross-sectional study assessing patients who underwent BP measurement in the TeleMRPA platform (www.telemrpa.com) from May 2017 to September 2019. The platform has been developed as a remote diagnosis tool for BP telemonitoring, and its characteristics allow the analysis and filtering of a database according to the scientific questions investigated. The mathematical algorithm used allows the protection of the patients' personal data, as well as of the data from the clinics or healthcare units, for the interpretation of the tests and the construction of research projects. Because it is not a software but a platform that can be accessed from any computer, tablet or smartphone, the input of BP measurement data can be performed remotely and in a simple way.

This study included patients aged 18 years and over, managed with antihypertensive drugs in a single-drug therapy strategy or a combination of two different classes of antihypertensive drugs. The exclusion criteria were as follows: use of no antihypertensive drug; combined use of three or more antihypertensive drugs; and use of spironolactone and alpha-2 agonist as a single-drug therapy (Figure 1).

**Figure 1:** *Flowchart of the study's sample selection*.

Source: the authors

The following data of the TeleMRPA platform were used: sex (male/female); age (in years, calculated from the birth date); body mass index (BMI); number of valid BP measurements at home; systolic and diastolic blood pressure (SBP and DBP, respectively) obtained from HBPM and casual measurement; and class of antihypertensive drug used.

To calculate BMI, weight and height were measured and the Quetelet's index used.^11^ On the first visit to the doctor's office, the patient received a BP device for home BP measurement in addition to instructions on its proper management.^3^ On that same day, two BP measurements were taken at the office and, in the following four days, the patient (and/or caregiver) took BP measurements at home according to a protocol. Casual BP measurement was defined as the mean of the two BP measurements taken at the office, while home BP measurement was defined as the mean of the 24 BP measurements taken from the second to the fifth day (Figure 2).^12^^,^^13^

**Figure 2:** Protocol of home blood pressure monitoring (HBPM) according to the Brazilian guidelines (***/**: blood pressure measurement).*8,9*

Validated automated Omron, Geratherm and Microlife devices were used.

Data were exported from the TeleMRPA platform to excel. All classes of drugs described in the platform were reviewed and encoded by two work teams. Then, the databases were crossed for identification of discrepancies, which, when present, were reviewed by the entire team and the coordination.

### Statistical analysis

For data analysis, the software Stata, version 14.0, was used. Descriptive statistics with means and standard deviation was used for normally distributed continuous variables, and absolute and relative frequencies, for categorical variables. Kolmogorov-Smirnov test was used to assess the data distribution of the variables. The BP levels obtained from HBPM and casual measurement were compared by using paired *t* test. To compare the BP levels according to the drugs used, unpaired *t* test was applied, while patients reaching and not reaching BP goals in casual measurement and HBPM were compared by using chi-square test. The 5% significance level was adopted for all analyses.

## Results

This study assessed 6731 patients, 61.3% of the female sex, mean age of 57.8 (±12.6) years, and mean BMI of 29.0 (±5.1) kg/m^2^. The BP levels taken casually were higher than those taken at home, and this behavior was seen with the use of single-drug therapy or of a combination of drugs, and for all drug classes (Table 1). The mean number of valid BP measurements was 23.5 (±1.6). The differences in the mean SBP and DBP levels were 6.6 mm Hg (p<0.001) and 4.4 mm Hg (p<0.001), respectively. Those differences characterize the white-coat effect and maintained statistical significance in all treatment strategies.

**Table 1:** Sample description according to the antihypertensive drug used, and comparison between the methods used to measure blood pressure, casual and home blood pressure monitoring (HBPM), total and according to drug strategy, n= 6731

Variable	n	%	HBPM	Casual	p
**Total**					
SBP	6731	100	126.2 ± 15.3	132.8 ± 19.6	<0.001
DBP			79.6 ± 9.7	84.0 ± 11.6	<0.001
**ARB**					
SBP	2254	33.5	127.1 ± 15.2	133.5 ± 19.2	<0.001
DBP			80.4 ± 9.6	84.9 ± 11.6	<0.001
**ACEI**					
SBP	595	8.8	124.7 ± 14.2	130.2 ± 18.1	<0.001
DBP			79.3 ± 9.0	83.0 ± 10.5	<0.001
**CCB**					
SBP	196	2.9	127.2 ± 13.1	134.0 ± 17.4	<0.001
DBP			80.2 ± 9.5	84.4 ± 11.2	<0.001
**Diuretic**					
SBP	173	2.6	123.1 ± 13.8	132.2 ± 19.1	<0.001
DBP			79.1 ± 9.3	85.5 ± 10.6	<0.001
**Beta-blocker**					
SBP	474	7.0	123.2 ± 15.2	130.6 ± 19.4	<0.001
DBP			77.7 ± 10.0	82.4 ± 11.2	<0.001
**ARB + CCB**					
SBP	683	10.1	127.1 ± 14.6	133.6 ± 19.1	<0.001
DBP			79.0 ± 9.7	83.2 ± 11.8	<0.001
**ACEI + CCB**					
SBP	332	4.9	126.2 ± 12.6	132.4 ± 16.3	<0.001
DBP			79.9 ± 8.9	83.8 ± 10.4	<0.001
**ARB + diuretic**					
SBP	1015	15.1	125.2 ± 16.2	132.8 ± 21.1	<0.001
DBP			79.6 ± 9.6	84.5 ± 12.1	<0.001
**ACEI + diuretic**					
SBP	151	2.2	124.6 ± 15.8	132.7 ± 20.0	<0.001
DBP			78.8 ± 9.5	84.0 ± 11.1	<0.001
**Beta-blocker + ACEI**					
SBP	134	2.1	127.6 ± 17.1	133.9 ± 21.9	<0.001
DBP			79.0 ± 10.4	82.4 ± 13.2	<0.001
Beta-blocker + ARB					
SBP	475	7.1	129.5 ± 18.0	135.5 ± 22.3	<0.001
DBP			79.1 ± 10.6	83.0 ± 12.7	<0.001
**Beta-blocker + diuretic**					
SBP	137	2.0	122.0 ± 14.9	130.9 ± 21.0	<0.001
DBP			78.1 ± 8.3	84.0 ± 11.3	<0.001
**Beta-blocker + CCB**					
SBP	65	1.0	125.5 ± 16.4	131.9 ± 21.6	<0.001
DBP			77.4 ± 10.7	82.1 ± 12.1	<0.001
**CCB + diuretic**					
SBP	45	0.7	130.8 ± 14.6	137.1 ± 18.6	<0.001
DBP			81.7 ± 11.0	86.3 ± 13.1	<0.001

*Paired t test. ARB: angiotensin receptor blocker; ACEI: angiotensin-converting-enzyme inhibitor; CCB: calcium channel blocker; SBP: systolic blood pressure; DBP: diastolic blood pressure. Source: the authors.*

Regarding the drug treatment strategy, 54.8% of the patients were on single-drug therapy and 45.2% were on combinations of two drugs. Renin-angiotensin-aldosterone system (RAAS) blockers were the most frequent option, and 58.7% of the patients were using angiotensin receptor blockers (ARB), while 15.9% were on angiotensin converting enzyme inhibitors (ACEI) (Table 1).

When assessing BP control according to the goals ‘lower than 140 mm Hg and 90 mm Hg’ for casual measurement, and ‘lower than 135 mm Hg and 85 mm Hg’ for HBPM, according to current guielines,^3^^,^^4^ our rates were 57.0% and 61.3%, respectively (p<0.001). The prevalence of UCWCH was 15.4% and that of UCMH, 11.1% (Table 2).

**Table 2:** Blood pressure (BP) control assessed via casual measurement (< 140 and <90 mm Hg) and home blood pressure monitoring (HBPM: < 135 and < 85 mm Hg), n= 6731

	BP goal in casual measurement		Total	
	<140 and <90 mm Hg	≥ 140 and/or ≥ 90 mm Hg		
BP goal in HBPM				
< 135 and < 85 mm Hg	3093 (45.9%)	1034 (15.4%)*	4127 (61.3%)	p< 0.001
≥ 135 and/or ≥ 85 mm Hg	744.9 (11.1%)†	1860 (27.6%)	2604 (38.7%)	
Total	3837 (57.0%)	2894 (43.0%)	6731 (100.0%)	

*Chi-square test.*

* *Uncontrolled white-coat hypertension,*

† *uncontrolled masked hypertension. Source: the authors.*

The comparisons of SBP and DBP obtained via HBPM according to the different classes of antihypertensive drugs are shown in Table 3 (single-drug therapy) and Table 4 (combinations of two drugs).

**Table 3:** Significance values (p) of the comparisons of systolic blood pressure and diastolic blood pressure obtained from home blood pressure monitoring, according to the different classes of antihypertensive drugs in single-drug therapy, n= 6731

Drugs					
	**SBP comparisons**	ACEI	CCB	DIUR	BB
ARB		**<0.01**	0.987	**<0.001**	<0.001
ACEI		-	**0.035**	0.060	0.095
CCB		-	-	**<0.001**	**0.002**
DIUR		-	-	-	0.630
BB		-	-	-	-
	**DBP comparisons**	ACEI	CCB	DIUR	BB
ARB		**0.009**	0.737	**<0.001**	**<0.001**
ACEI		-	0.231	0.115	0.005
CCB		-	-	**0.028**	**0.002**
DIUR		-	-	-	0.557

*Unpaired t test. ARB: angiotensin receptor blocker; ACEI: angiotensin-converting-enzyme inhibitor; CCB: calcium channel blocker; DIUR: diuretic; BB: beta-blocker; SBP: systolic blood pressure; DBP: diastolic blood pressure. Source: the authors.*

**Table 4:** Significance values (p) of the comparisons of systolic blood pressure and diastolic blood pressure obtained from home blood pressure monitoring according to the different combinations of two antihypertensive drugs, n= 6731

Combination of 2 drugs								
SBP comparisons	ACEI with CCB	ARB with DIUR	ACEI with DIUR	BB with ACEI	BB with ARB	BB with DIUR	BB with CCB	CCB with DIUR
ARB with CCB	0.358	**0.018**	0.070	0.713	0.012	**<0.001**	0.419	0.099
ACEI with CCB	-	0.317	0.239	0.332	**0.004**	**0.001**	0.696	**0.023**
ARB with DIUR	-	-	0.671	0.117	**<0.001**	**0.026**	0.894	**0.024**
ACEI with DIUR	-	-	-	0.132	**0.003**	0.144	0.714	0.021
BB with ACEI	-	-	-	-	0.276	0.004	0.417	0.263
BB with ARB	-	-	-	-	-	**<0.001**	0.093	0.641
BB with DIUR	-	-	-	-	-	-	0.129	**<0.001**
BB with CCB	-	-	-	-	-	-	-	0.087

DBP comparisons	ACEI with CCB	ARB with DIUR	ACEI with DIUR	BB with ACEI	BB with ARB	BB with DIUR	BB with CCB	CCB with DIUR
ARB with CCB	0.133	0.195	0.800	0.995	0.842	0.270	0.204	0.077
ACEI with CCB	-	0.581	0.190	0.319	0.243	**0.030**	**0.040**	0.239
ARB with DIUR	-	-	0.317	0.485	0.366	0.065	0.072	0.166
ACEI with DIUR	-	-	-	0.856	0.725	0.475	0.343	0.087
BB with ACEI	-	-	-	-	0.903	0.397	0.313	0.145
BB with ARB	-	-	-	-	-	0.264	0.216	0.125
BB with DIUR	-	-	-	-	-	-	0.644	**0.020**
BB with CCB	-	-	-	-	-	-	-	**0.043**

*Unpaired t test. ARB: angiotensin receptor blocker; ACEI: angiotensin-converting-enzyme inhibitor; CCB: calcium channel blocker; DIUR: diuretic; BB: beta-blocker; SBP: systolic blood pressure; DBP: diastolic blood pressure. Source: the authors.*

## Discussion

This study contributes to the analysis in clinical practice of a significant number of hypertensive patients undergoing drug treatment, assessed by use of casual BP measurement and HBPM. Our findings confirm that lower mean BP levels are observed in HBPM regardless of the use of single-drug therapy or drug combination or even of the class of the antihypertensive drug prescribed. A high rate of BP control was found, higher with HBPM, in addition to a significant prevalence of UCWCH and UCMH, with potential prognostic implications, reinforcing the need for out-of-the-office BP measurement as a parameter for proper management and follow-up of hypertensive patients.

Regarding the characteristics of the sample studied, it is worth emphasizing the population's mean age close to 60 years and increased BMI, which are relevant aspects that make SAH treatment as well as SAH control more difficult.^14^^–^^16^

In addition, although the latest national and international guidelines recommend the combination of drugs as the preferred strategy for most hypertensive patients, 54.8% of our sample were on single-drug therapy.^3^^,^^4^^,^^17^^,^^18^ The analysis of the classes of antihypertensive drugs used evidenced the preference for the RAAS blockade strategy, which was observed in 74.6% of the patients, and a 3.7-time greater frequency of ARB use as compared to ACEI use. Other classes of antihypertensive drugs had a low frequency of use in single-drug therapy. The combination of ARB or ACEI with diuretics (DIUR) or calcium channel blockers (CCB) was preferred, which is in accordance with the current recommendations.^19^^,^^20^

The SBP and DBP levels were always higher and statistically significant in the measurements taken in the office than at home, regardless of the antihypertensive drug class and of the single-drug or combination strategy. On average, the differences were 6.6 mm Hg for SBP (p<0.001) and 4.4 mm Hg for DBP (p<0.001), and these differences remained at a higher or lower intensity for all drugs used. This evidences the need to consider the UCWCH as a frequent phenotype among patients treated, which can induce the use of higher doses of drugs than required when assessing hypertensive patients treated based only on BP measurements taken at the office.^2^^,^^8^^,^^9^^,^^21^ In our sample, the prevalence of UCWCH was 15.4% and that of UCMH, 11.1%, meaning a 26.5% misinterpretation of proper BP control with the therapeutic strategy.

The UCWCH is even more relevant if we consider that, from the HBPM perspective, more patients reached the BP goal as compared to those assessed via casual measurement (61.3% vs 57%, p<0.001), reinforcing the thesis that, with home measurements, more proper adjustments to BP levels can be obtained daily. It is worth considering that, differently from what we imagined, UCWCH can be found in elevations of the SBP or DBP component via casual measurement and in all age ranges.^21^ Another phenotype to be considered is UCMH, still one of the major doubts in SAH treatment: how to manage patients with controlled BP at the office but with elevated BP levels taken at home, and which would be the BP goals for HBPM?^22^

When comparing the classes of antihypertensive drugs, significant differences were found regarding the reduction in BP levels. However, one limiting factor should be considered: because this study was not randomized for the comparison of antihypertensive drug classes, the doses used and the patients' characteristics, those differences should be carefully analyzed. Those findings are in accordance with those of other publications, mainly meta-analyses of randomized studies, which had already described differences in drug potency depending on the doses used and indications but based on casual BP measurement.^23^^–^^25^ In this study, the different classes of antihypertensive drugs seem to have different potencies when assessed via HBPM, reinforcing the need for patient's individualization when choosing the best treatment strategy.

From our viewpoint, a relevant aspect in this study is the higher prevalence of ARB use as the strategy for RAAS blockade and its combination with CCB and DIUR. The preference for RAAS blockade to treat SAH might have been based on the literature evidence of significant BP reduction, cardiovascular protection, and low incidence of side effects with the use of that drug class, in both single-drug and combination therapy.^26^^,^^27^

## Conclusions

For hypertensive patients undergoing treatment, the proper BP control assessment should be based not only on measurements taken at the office but at home as well.

The different classes of antihypertensive drugs behave differently regarding BP reduction even for HBPM, and this finding should be further investigated in prospective randomized trials.

The high prevalence of UCWCH and UCMH found suggests that, when the therapeutic decision is based only on measurements taken at the office, the BP management might be inappropriate, with a potential impact on the management and follow-up of hypertensive patients.

### National co-investigators

Adriana Camargo Oliveira (Goiânia, GO), Adriana Siqueira S. Menezes (Recife, PE), Ana Paula Nana P Martingo (Sorocaba, SP), Anderson da Costa Armstrong (Petrolina, PE), Anderson Daniel C. Rodrigues (São José dos Campos, SP), Andrea Garcez R. Dias (Rio de Janeiro, RJ), André K. Vidigal de Vasconcellos (Caruaru, PE), Ângela Maria F. Lima (Gravataí, RS), Annelise Machado Gomes de Paiva (Maceió, AL), Anselmo Honorato S. Souza (Goiânia, GO), Antônio Almeida Braga (Recife, PE), Anyotan Cruz Nascimento (Americana, SP), Aristeu Haroldo K. Mizuta (São José dos Campos, SP), Breno Gontijo de Camargos (Taguatinga, DF), Bruno Alencar Fonseca (Belo Horizonte, MG), Bruno Augusto A. Nogueira (São José dos Campos, SP), Bruno Daniel Ferrari (Assis, SP), Bruno José P. Coutinho (Carpina, PE), Carlos Alberto Machado (Campos do Jordão, SP), Carlo Bonasso (São Paulo, SP), Carlo Bonasso Filho (São Paulo, SP), Carlos Filinto de Almeida (Campo Grande, MS), Carolina Paes B. Saturnino Braga (Rio de Janeiro, RJ), César Lourenço Nezello (Lagoa Vermelha, RS), Claudinelli Alvarenga Aguilar (Goiânia, GO), Cristiane Bueno de Souza (Campos do Jordão, SP), Daniella Rosano Gregorini (Jundiaí, SP), Danielle Batista Leite (Fernando Figueira, PE), Eduardo Azevedo Junior (Cabo Frio, RJ), Eduardo Siqueira (Mogi das Cruzes, SP), Eduardo Zen (Curitiba, PR), Elder Gil Alves da Cruz (Salgueiro, PE), Eliel Barreto César (Americana, SP), Erika Maria Gonçalves Campana (São Gonçalo, RJ), Esther G.D. Lima de Barros Carvalho (Guarabira, PB), Euler Manenti (Porto Alegre, RS), Fabiano de Souza Ramos (Nova Iguaçu, RJ), Fábio Argenta (Cuiabá, MT), Fábio Serra Silveira (Aracaju, SE), Fernando Alfredo Fonseca (Petrópolis, RJ), Fernando Pivatto (Porto Alegre, RS), Flávia Karina S. Oliveira (São José dos Campos, SP), Flávio Henrique A. Pires Véras (Mossoró, RN), Francisco Deoclécio Pinheiro (Itapipoca, CE), Giovanni Alves Saraiva (Olinda, PE), Guilhermo Hinestrosa (São Paulo, SP), Gustavo A.C.S. Barros (Recife, PE), Gustavo Costa Motta (Santa Maria, RS), Hiran de Paula (Itabaiana, SE), Humberto Graner Moreira (Goiânia, GO), Idália de Sousa Andrade (São Paulo, SP), Ivaldo Calado (Recife, PE), Jadil Francisco Fusturath Jr (Porto Velho, RO), Joaquim Carlos P. Sales (Ribeirão Preto, SP), João Felix Morais Filho (Natal, RN), João Francisco M. Pacheco (Belém, PA), João Henrique R. Ferreira (Gama, DF), Jonathan Scapin Zagatti (Jales, SP), José Roberto B. Leal Filho (Caruaru, PE), Josafá de Oliveira Costa (Igarassu, PE), José Roberto Moya (Cuiabá, MT), José Wladimir Tambelli Pires (Itapetinga, SP), Josiedson Pontes de Farias (Caruaru, PE), Leonardo da Costa Velasco (Campos dos Goytacazes, RJ), Lilian Mesquita (Rio de Janeiro, RJ), Lola Santos F. Quinta (Goiânia, GO), Lorena Maia (Goiânia, GO), Luam Vieira de Almeida Diógenes (Teresina, PI), Lúcia Cristina F. Lenzi (Rio de Janeiro, RJ), Luís Fernando M. Mandrá (Americana, SP), Luiz Kencis Junior (São Paulo, SP), Luiz Fernando de Oliveira Urzeda (Goiânia, GO), Marcelo De Carli Cavalcanti (Petrolina, PE), Marcelo Júlio de Oliveira (Ribeirão Preto, SP), Marco Antônio Alves (Escada, PE), Marco Antônio C. Peralva (Juiz de Fora, MG), Maria Beatriz M.B. Rodrigues (Porto Velho, RO), Maria Christina C. Ballut (Manaus, AM), Murillo Antunes (Bragança Paulista, SP), Nelson Dinamarco (Ilhéus, BA), Nildo Magalhães (Jundiaí, SP), Otacílio Gomes de Oliveira Filho (Sete Lagoas, MG), Paulo E.F. Ortiz Júnior (Porto Alegre, RS), Paulo Meirelles (Salvador, BA), Paulo Roberto P. Sant'Ana (Nova Iguaçu, RJ), Paulo Sergio L. Soares (Vassouras, RJ), Pedro Guimarães M. Silva (Goiânia, GO), Rafael Nogueira de Macedo (Fortaleza, CE), Rafael Santos Costa (Nova Iguaçu, RJ), Reginaldo Peixoto de Melo Neto (Recife, PE), Roberto Dultra (Itabuna, BA), Robson Pierre Pacífico A. Filho (Goiânia, GO), Rodrigo Cunha (Uberaba, MG), Rogério Krakauer (Santo André, SP), Rogério M. Ruiz (São Paulo, SP), Rosa Maria Rondon (Campo Grande, MS), Tássia Tâmara Silva Feitosa (Recife, PE), Rui Morando (Americana, SP), Tsuneo Antônio Alberto Goto (Guarulhos, SP), Valéria Tatyane de Rezende (Goiânia, GO), Vanderlei Magalhães da Silveira (Passo Fundo, RS), Vanildo S. Guimarães Neto (Recife, PE), Victoria Alves Melo (Goiânia, GO), Vilma Helena Burlamaqui (Niterói, RJ), Wenderson Tavares dos Santos (Belo Horizonte, MG).
